# CLOVES Syndrome in a Nine-month-old Infant

**DOI:** 10.7759/cureus.5772

**Published:** 2019-09-26

**Authors:** Sara Alomar, Rewana E Khedr, Saad Alajlan

**Affiliations:** 1 College of Medicine, Alfaisal University, Riyadh, SAU; 2 Dermatology, King Faisal Specialist Hospital and Research Centre, Riyadh, SAU

**Keywords:** pik3ca-related overgrowth spectrum, cloves syndrome, pik3ca

## Abstract

CLOVES syndrome is a recently described overgrowth syndrome. Clinically, it is characterized by congenital lipomatous overgrowth (CLO), vascular anomalies (V), epidermal nevi (E), and skeletal deformities (S). Genetically, it is characterized by a somatic gain-of-function mutation of the phosphatidylinositol-4,5-bisphosphate 3-kinase catalytic subunit alpha (PIK3CA) gene. This somatic mutation is, in turn, associated with the activation of the protein kinase B-mammalian target of the rapamycin (AKT-mTOR) pathway that drives various signaling cascades. The end result is eventually promoting cell proliferation, growth, and survival. CLOVES syndrome is exceedingly uncommon, with less than 200 cases currently documented. Herein, we describe a case of CLOVES syndrome in a nine-month-old male infant who was referred to our dermatology clinic for further assessment and management. The diagnosis was made based on clinical findings and confirmed by genetic testing.

## Introduction

Phosphatidylinositol-4,5-bisphosphate 3-kinase catalytic subunit alpha (PIK3CA)-related overgrowth spectrum (PROS) defines a varied group of infrequent disproportionate overgrowth conditions prompted by post-zygotic variants in the PIK3CA gene located in the 3q26.32 chromosome [1]. CLOVES syndrome is one example of PROS, and its clinical features have been previously defined by Sapp et al. [2] in 2007 and expanded by Alomari [3] in 2009. CLOVES syndrome exhibits unique clinical findings, and it stands for congenital lipomatous overgrowth (CLO), vascular anomalies (V), epidermal nevi (E), and skeletal deformities (S). CLOVES syndrome is an extremely rare disorder; less than 200 cases have been reported so far [4-5]. Due to the extremely low prevalence rate of CLOVES syndrome, not much epidemiological data are available in the literature. Nevertheless, CLOVES syndrome is characterized by a congenital (early) childhood onset. It appears to affect males and females equally, irrespective of ethnicity, and its incidence rate is estimated to be less than 1:1,000,000. Herein, we present the case of CLOVES syndrome in a nine-month-old male patient who was referred from a local hospital to the dermatology clinic at King Faisal Specialist Hospital and Research Centre (KFSH&RC), Riyadh, Saudi Arabia.

## Case presentation

A nine-month-old male infant presented with lipomatous overgrowth of the right side of the abdomen, as well as swelling and scattered skin pigmentation over the right lower limb. The parents indicated that these findings were present since birth; however, they grew progressively over time. The infant was up-to-date with his vaccinations and had normal growth and mental developmental milestones for his age. Past medical history was notable for recurrent serious infections since birth, which were attributable to the underlying vascular-lymphatic malformations.

**Figure 1:**
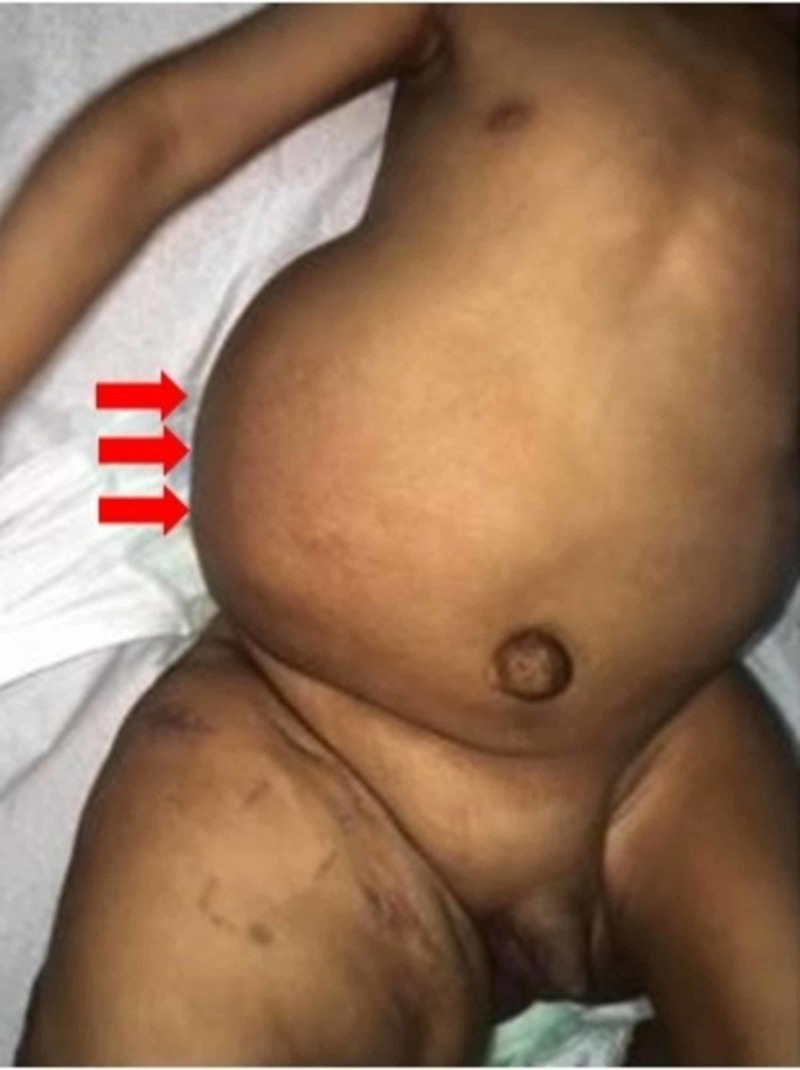
Lipomatous overgrowth is noted over the right side of the abdomen (red arrows)

**Figure 2:**
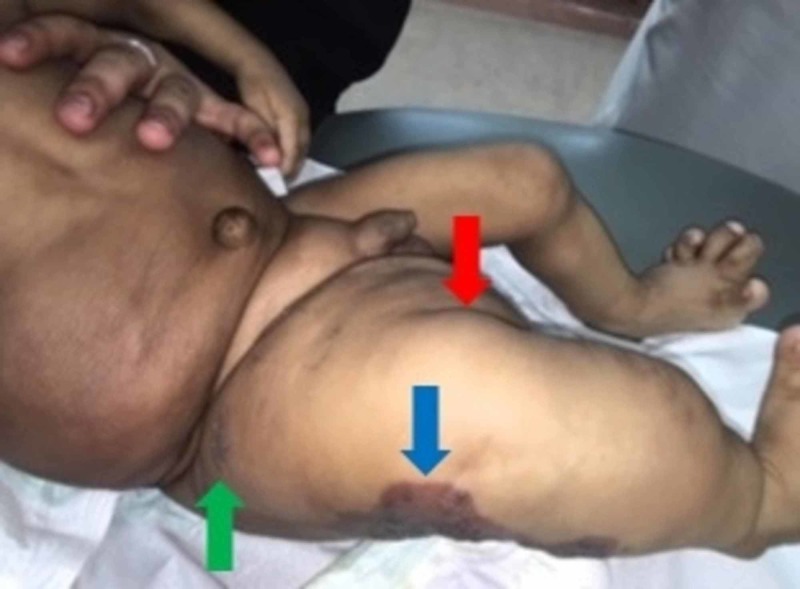
Lipomatous overgrowth (red arrow), epidermal nevus (green arrow), and pigmented skin lesion mostly suggestive of capillary malformation (blue arrow) are noted over the right lower limb

**Figure 3:**
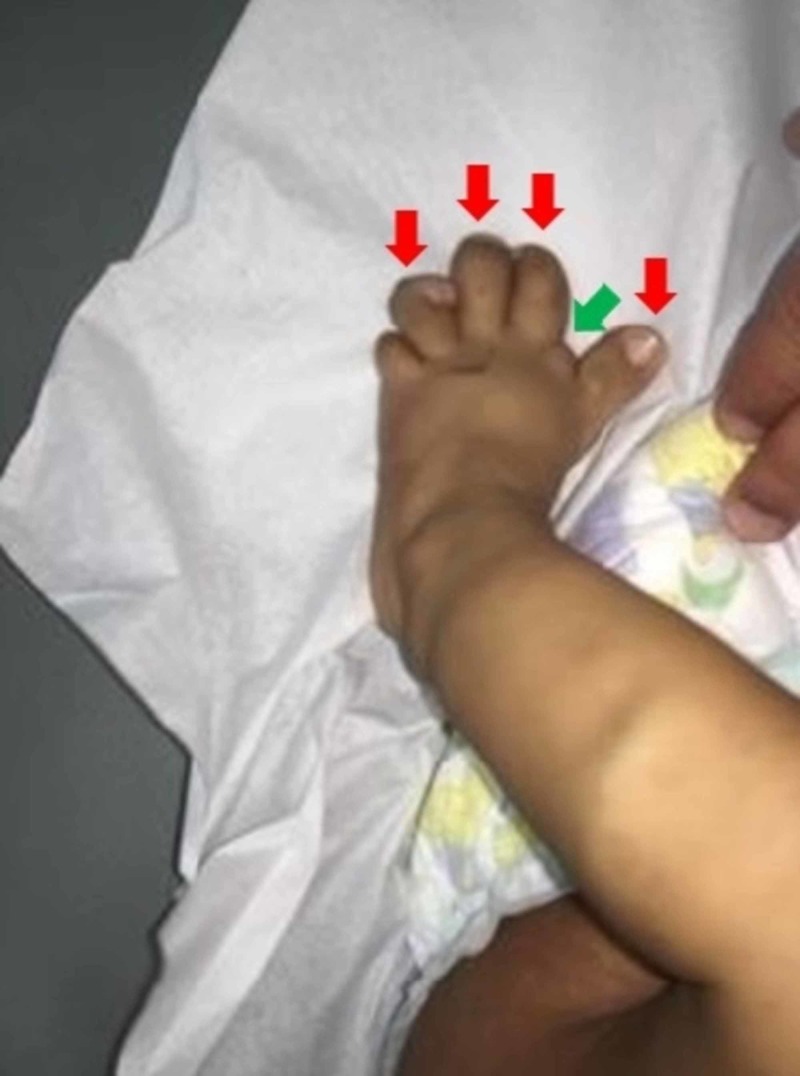
Bone deformity (green arrow) and macrodactyly (red arrows) are noted over the left foot

Upon general examination, the infant was playful and active. Physical examination was notable for an enlarged right-sided abdomen, mostly of fat-like density without organomegaly (Figure 1). Moreover, the right lower limb showed lipomatous overgrowth, epidermal nevi, and a pigmented skin lesion mostly suggestive of capillary malformation (Figure 2). Additionally, both feet showed bone deformities and macrodactyly (Figure 3).

**Figure 4:**
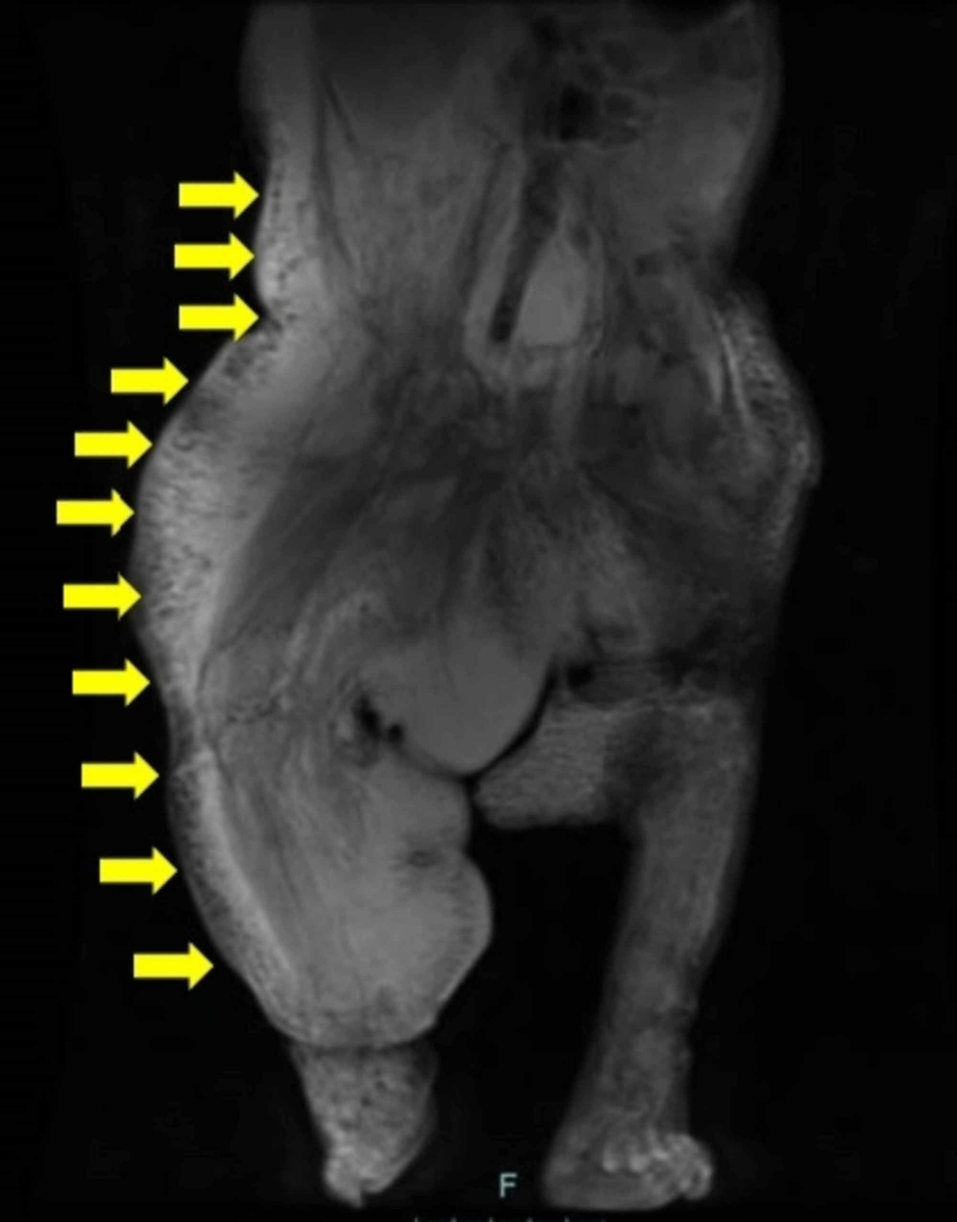
Pre-gadolinium T2-weighted coronal magnetic resonance imaging (MRI) scan showed white lymphatic malformations in most of the right side of the body (yellow arrows)

**Figure 5:**
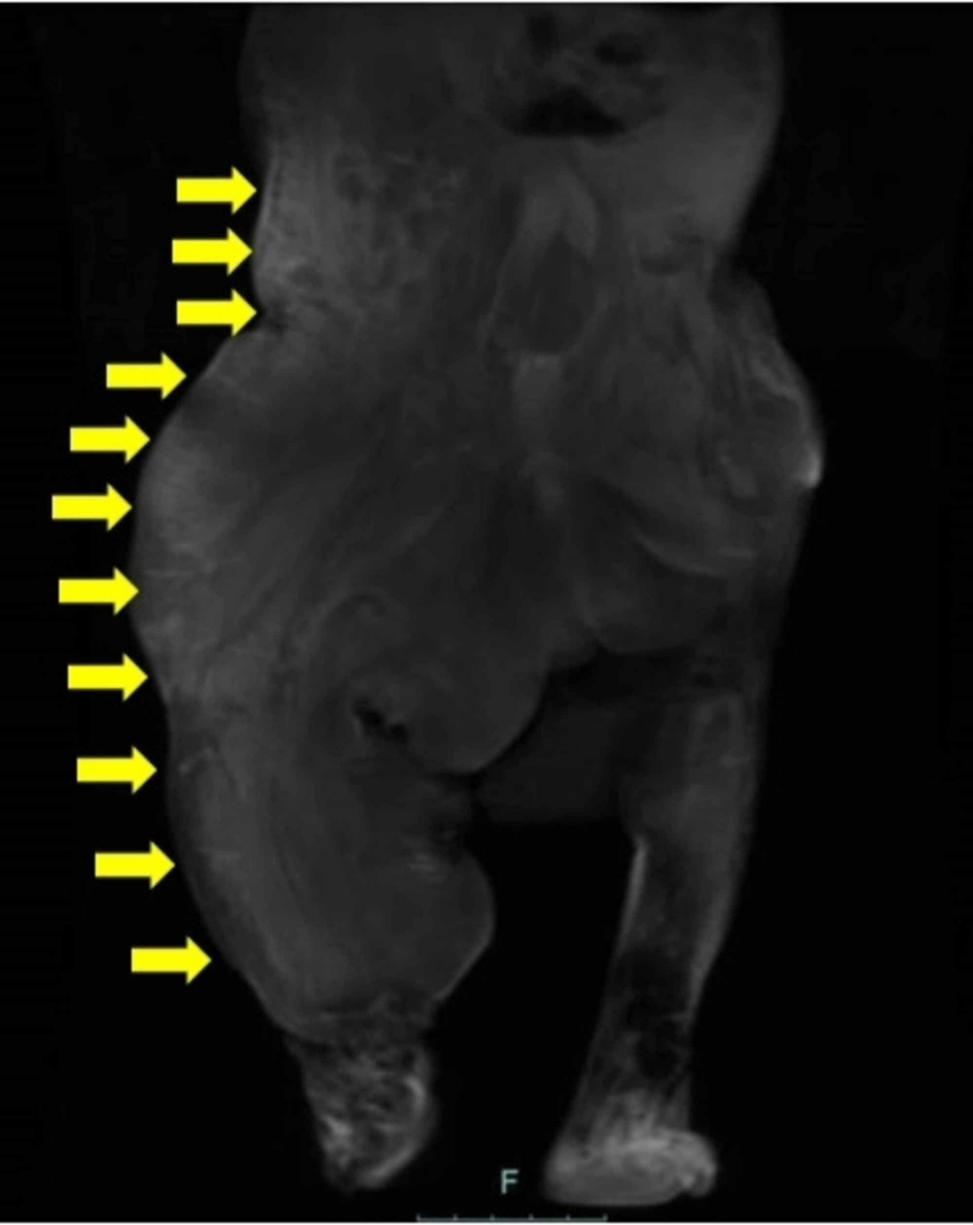
Post-gadolinium T1-weighted coronal magnetic resonance imaging (MRI) scan showed non-enhanced (black) lymphatic malformations in most of the right side of the body (yellow arrows)

Pre-gadolinium (Figure 4) and post-gadolinium (Figure 5) magnetic resonance imaging (MRI) showed soft tissue hypertrophy along with veno-lymphatic malformations involving the right abdomen and lower extremity.

Differential diagnosis of overgrowth syndrome was suggested. The parents consented for tissue biopsy and genetic testing. In-house genetic sequencing of normal and abnormal tissue (epidermis-hypodermis) from the thigh demonstrated a mutation of the PIK3CA gene, and thus a diagnosis of CLOVES syndrome was established. The procedure was done as per the hospital's protocol, and one sequence from each normal and abnormal tissue was examined.

The parents were educated about sirolimus for the management of the predominant vascular malformations, and signed consent was obtained. Sirolimus was introduced at a target concentration level of 2.1 mg/m^2^/day. However, one day later, the patient presented to the emergency department with high-grade fever and was diagnosed with urinary tract infection (UTI). The clinical decision was to stop sirolimus and start a two-week course of ceftazidime. After two weeks, the infection subsided and the patients’ clinical status improved, thus, sirolimus was resumed again. At one-month follow-up at the dermatology clinic, the sirolimus level was found to be 4.5 µg/L and a shrinkage in the size of the lesion was noted. The dose was increased to 2.2 mg/m^2^/day, and the patient was scheduled for a follow-up visit after six months. Five months later, the parents noted high spikes in the patient’s temperature, and on their way to the hospital, the patient passed away.

## Discussion

The differential diagnoses of overgrowth syndromes are numerous, for example, CLOVES syndrome, fibroadipose overgrowth (FAO), Proteus syndrome, Klippel-Trénaunay syndrome, and Sturge-Weber syndrome [6]. A definitive diagnosis of CLOVES syndrome can be established by genetic testing to guarantee the identification of the CLOVES syndrome-specific underlying mutations [4,7]. For example, both CLOVES syndrome and FAO harbor mutations in the PI3KCA gene; however, a mutation in the C2 domain of the PI3KCA gene can, to a greater extent, differentiate CLOVES syndrome from FAO [6]. On the other hand, Proteus syndrome and lipodystrophy syndrome-hypoglycemia are characterized by activating somatic mutations of the AKT1 and AKT2 genes, respectively [6]. Unfortunately, facilities for genetic sequencing may be only accessible at specialized healthcare institutions. Moreover, CLOVES-specific mutations are poorly expressed in blood samples [8]. Thus, genetic sequencing generally demands an evaluation of at least one tissue sample from both the affected and unaffected (control) tissues. Since these overgrowth syndromes are characterized by somatic variants in the genes associated with PI3K/AKT/mammalian target of rapamycin (mTOR), Sanger sequencing may prove ineffective and yield negative results in light of the mosaic nature of these variants [9-10]. Recently, Chang et al. [10] demonstrated that targeted next-generation sequencing (NGS) technology can permit (with high sensitivity) identification of the low-level mosaicism, and thus enable the diagnosis of mosaic overgrowth syndromes in both prenatal and postnatal settings. If CLOVES syndrome diagnosis cannot be established by genetic sequencing or NGS, then the diagnosis can be established clinically [7]. Patients with CLOVES syndrome, to a larger degree, are free of hematopoietic, cardiovascular, and gastrointestinal anomalies [11]. Although almost all patients with CLOVES syndrome are intellectually normal, one report by Alomari showed that neurologic impairment was observed in around 50% of the study cohort [12]. Thorough details of overgrowth syndromes, generally, and PROS, specifically, are reviewed in the literature [6,13]. Table 1 displays the major clinical features of PROS [6].

**Table 1:** Major clinical features of PIK3CA-related overgrowth spectrum (PROS) Information was obtained from the following citation: Keppler-Noreuil KM, Rios JJ, Parker VE, et al.: PIK3CA-related overgrowth spectrum (PROS): diagnostic and testing eligibility criteria, differential diagnosis, and evaluation. Am J Med Genet A. 2015, 167A:287-295. 10.1002/ajmg.a.36836.

Overgrowth disorder	Clinical features
Macrodystrophia lipomatosa	Fibrofatty tissue enlargement and bony overgrowth, typically within a ‘nerve territory’ with enlargement in circumference and length of the peripheral nerve
Hemihyperplasia with multiple lipomatosis (HHML)	Macrodactyly, moderate abnormalities of asymmetry and overgrowth with multiple subcutaneous lipomata, hemihyperplasia may be static or mildly progressive
Fibroadipose overgrowth (FAO)	Segmental and progressive overgrowth of subcutaneous and visceral fibro-adipose tissue, sometimes associated with skeletal and muscular overgrowth
Facial infiltrating lipomatosis	Hemifacial soft-tissue and skeletal overgrowth, precocious dental development, macrodontia, hemimacroglossia, mucosal neuromas
CLOVES syndrome	Congenital lipomatous overgrowth, vascular anomalies, epidermal nevi, skeletal deformities
Megalencephaly-Capillary Malformation (MCAP, or M-CM)	Megalencephaly, ventriculomegaly that may progress to hydrocephalus, cerebellar tonsillar ectopia that may progress to Chiari malformation, cortical brain abnormalities
Muscular hemihypertrophy	Muscular overgrowth on one side of the body

The management of CLOVES syndrome is quite challenging, and consensus management guidelines for CLOVES syndrome are poorly described in the literature [8]. Generally, there is no definitive cure for CLOVES syndrome, and management is primarily geared toward improving the quality of life. Surgical debulking for lipomatous masses can be technically challenging, associated with high morbidity and high rates (~ 100%) of postoperative recurrence [12]. Thus, the decision of surgical intervention demands both parties (that is, the parents and the surgeon) to thoughtfully weigh the risks-benefits prior to embarking on the surgical intervention.

The vascular anomalies implicated in CLOVES syndrome can present substantial morbidity and mortality to the patient. Pharmacologic mammalian target of rapamycin (mTOR) inhibitors, such as sirolimus, have been shown to exhibit therapeutic responses with acceptable toxicity profiles in patients with complex vascular anomalies [14]. In a recent review of 150 patients with complex vascular anomalies treated with sirolimus, efficacy was demonstrated in 85% of patients and only 3.33% (n=5) of patients achieved complete resolution. Critical inquiries about sirolimus continue to be poorly answered in the literature, such as: what is the most proper dosage and for how long the patient should be treated [15]. In our study, the reason for the patient's death is attributable to the long-standing history of recurrent serious infections (due to the vascular-lymphatic malformations) rather than sirolimus therapy. More recently, targeted therapy with an inhibitor of PIK3CA (BYL719) demonstrated effective vascular-related responses in 19 patients with PROS harboring complex vascular malformations [16]. BYL719 therapy was free of substantial drug-related adverse events. However, BYL719 requires further validation in large-scale PROS patients with complex vascular anomalies before withdrawing solid conclusions. Of note, BYL719 has entered phase I-II clinical trials to evaluate its efficacy and safety in patients with various PIK3CA-implicated solid tumors [17-19]. Recently, López Gutiérrez et al. [20] reported a 17-year-old girl with CLOVES syndrome who had large vascular malformations involving the external genitalia. The patient was initially administered oral rapamycin for 12 months. However, the patient did not exhibit substantial improvement with rapamycin and was subsequently started on low-dose BYL719. Within one month of treatment with BYL719, the patient demonstrated a remarkable response to therapy in terms of a reduction in the size of the vascular malformations, reduced vaginal bleeding, and improved quality of life. Due to the therapy-related downstaging of the vascular malformations, the patient underwent surgical debulking and reconstruction of her external genitalia. No therapy-related side effects were noted up to five months since the start of BYL719.

## Conclusions

CLOVES syndrome is extremely rare, and research in this area is largely confined to very scare published literature. Herein, we reported the first case of CLOVES syndrome from Saudi Arabia. Although clinical features can hint at the diagnosis of CLOVES syndrome, genetic testing for PIK3CA gain-of-function mutation establishes the definitive diagnosis. There is no absolute cure for CLOVES syndrome, and management is primarily geared toward improving the quality of life.
